# Aircraft Noise Distribution as a Fairness Dilemma—A Review of Aircraft Noise through the Lens of Social Justice Research

**DOI:** 10.3390/ijerph18147399

**Published:** 2021-07-11

**Authors:** Dominik Hauptvogel, Susanne Bartels, Dirk Schreckenberg, Tobias Rothmund

**Affiliations:** 1 German Aerospace Center, Institute of Aerospace Medicine, Sleep and Human Factors Research, Linder Höhe, 51147 Cologne, Germany; susanne.bartels@dlr.de; 2 Work and Engineering Psychology Research Group (Forschungsgruppe Arbeits- und Ingenieurpsychologie), Department of Human Sciences, Darmstadt University of Technology, Alexanderstr. 10, 64283 Darmstadt, Germany; 3 ZEUS GmbH, Zentrum für Angewandte Psychologie, Umwelt- und Sozialforschung, Sennbrink 46, 58093 Hagen, Germany; schreckenberg@zeusgmbh.de; 4 Institute for Communication Science, Friedrich-Schiller University Jena, Ernst-Abbe Platz 8, 07743 Jena, Germany; tobias.rothmund@uni-jena.de

**Keywords:** fairness, justice, procedural, distributional, informational, interpersonal, aircraft noise, aviation, annoyance

## Abstract

Aircraft noise exposure is a health risk and there is evidence that noise annoyance partly mediates the association between noise exposure and stress-related health risks. Thus, approaches to reduce annoyance may be beneficial for health. Annoyance is influenced by manifold non-acoustic factors and perceiving a fair and trustful relationship between the airport and its residents may be one of them. The distribution of aircraft noise exposure can be regarded as a fairness dilemma: while residents living near an airport may seem to have some advantages, the majority of residents living under certain flight routes or in their immediate proximity suffer from the disadvantages of the airport, especially the noise. Moreover, a dilemma exists between the airport’s beneficial economic impact for a region and the physical and psychological integrity of residents. Aircraft noise exposure through the lens of social justice research can help to improve our understanding of noise annoyance. Research indicates that the fairness perceptions of the parties involved can be enhanced by (a) improving individual cost–benefit ratios, (b) providing a fair procedure for deciding upon the noise distribution, and (c) implementing fair social interaction with residents. Based on the review of evidence from social justice research, we derive recommendations on how fairness aspects can be integrated into aircraft noise management with the purpose of improving the relationship between the airport and its residents, to reduce annoyance, and to enhance the acceptance of local aviation and the airport as a neighbor.

## 1. Introduction

Exposure to aircraft noise has been associated with a variety of different adverse health outcomes [1]. These range from annoyance due to noise [2,3,4,5,6], sleep disturbance during the night [7,8,9,10,11], associations with cardiovascular diseases [12], myocardial infarction [13], coronary heart disease [14], and blood pressure [15]. The harmful effect of noise has already been demonstrated for infants and children, on health, perception, and learning, e.g., through the deterioration of reading and oral comprehension in school children [16,17,18,19].

Annoyance due to noise is widespread in airport communities and occurs even at relatively low noise levels [2]. The concept of annoyance is multi-dimensional and comprises cognitive, emotional, and behavioral aspects [2]. Annoyance is not only regarded as a primary adverse effect of aircraft noise, but is assumed to be part of the causal pathway of other health outcomes and thus seems to mediate the effect of aircraft noise exposure and health risks [20]. Studies suggest that people reporting high levels of annoyance to have a higher risk for hypertension [21,22,23], a decrease in reported physical well-being [24], higher psychological distress [25], and, finally, an association was found between noise annoyance and the use of medication to treat anxiety disorders [26]. In addition, noise annoyance was observed to have an effect on sleep quality [5] and physical activity [27]. In other studies, aircraft noise did not have a direct effect on factors related to mental-health-related quality of life [28] or diagnoses of depression [29], but an indirect effect via annoyance was found. That means that the absolute noise exposure per se does not directly decrease mental health-related quality of life or diagnoses of depression, but perceiving annoyance does. Regarding mental health, one study revealed that the noise profile around an airport could have an effect, indicating that, for example, bigger airports with more flights including night flights affect mental health to a larger extent than noise profiles from smaller airports [30]. Furthermore, it was found that the degree of changes in noise exposure at airports also lead directly to a poorer mental health-related quality of life [28]. This indicates that situations of change (e.g., an expansion of an airport) are especially critical.

The mediating effect of annoyance on other health aspects can be explained by acknowledging that annoyance is a stress response. The sounds of aircraft only become noise when they are subjected to a certain evaluation. Aircraft noise can cause disturbance when trying to concentrate, for example. Therefore, aircraft noise, or any other environmental noise, can be seen as a stress factor [31]. In evolutionary terms, stress is a reaction necessary for survival that is triggered when we are faced with a dangerous situation, such as a wild animal. In modern times, however, these stress reactions are rarely triggered by life-threatening events. Nevertheless, in the human body, stress triggers reactions such as the release of cortisol or a change in blood pressure, which can have long-term health consequences [32]. However, the evaluation of a sound as noise is highly subjective and depends on a variety of non-acoustic factors, such as attitudes, expectations, and situational and personal factors [33]. Psychological models of noise reactions such as the model proposed by Stallen [34] suggest that the stress reaction, here the degree of annoyance, also depends on the possibility to cope with and control the stressor. Whether an individual perceives the ability to control the noise and the capacity to cope with it likewise depends on non-acoustic factors such as experienced trust in the authorities of the noise source, the predictability of the noise occurrence, influence on the noise source and access to information [34].

A major psychological construct implicitly underlying these non-acoustic factors is social (in)justice or (un)fairness (the two terms are used interchangeably throughout the manuscript). This construct has been extensively examined in the organizational and justice context with regard to the acceptance of outcomes of social exchange. In the present paper, the distribution of aircraft noise exposure and the relation between the noise source (i.e., the airport management) and the noise-affected individuals are reviewed from the perspective of fairness research, and it is thus considered to be a fairness dilemma. By adopting this perspective, the paper derives approaches for the reduction of adverse responses to noise by enhancing the perceived capacity to cope with noise in the affected individuals.

The construction or expansion of an airport often induces fears concerning the impairment of quality of life in many airport residents. European examples include the opening of the fourth runway at Frankfurt Airport, Germany, in 2011; the planned expansion of Heathrow Airport, UK, in 2026; and Florence Airport in 2029 [35,36], or the planned construction of a new terminal in Paris-Charles-de-Gaulle in 2021 [37]. An expansion, such as a new terminal or runway, usually comes along with an increase in flight movements, or at least a reshuffling of departures and/or approach routes and, thus, a redistribution of the noise. As a result, some residents will experience an increase in noise exposure. Thus, conflicts between the airport or airport stakeholders and the residents, as well as between residents from different communities, are predominantly connected to a shift of noise exposure.

As aircraft noise, unlike other types of noise (such as natural sounds), is caused by humans, it can be seen as a kind of social exchange [38,39]. Individuals (in this case (airport) operators) can be seen as responsible for the noise residents have to bear. The noise from aircraft can therefore be seen as a constant reminder of unfair treatment [38]. While there seem to be some advantages, for example, easy access to travel or potential employment opportunities for residents living around the airport, residents living under air corridors have to bear the noise with all its ramifications described above, as well as other costs, i.e., the loss of property value [40,41,42]. The uneven spread of noise in proximity to an airport area can be seen as a fairness dilemma: the noise has to be shouldered by one group, and the potential advantages of the airport are shared by others. Therefore, the ratio between the benefits and drawbacks of the nearby airport varies considerably between residents. Importantly, residents perceive having little control over the decision of how the burden of noise is distributed. Rothmund, Baumert and Zinkernagel [43] point out that the feeling of injustice can trigger strong emotional reactions and is therefore a motivational source for political protest and opposition, explaining the sometimes outraged and protesting residents living around the airport.

The present paper is addressed to noise researchers, airport authorities, and policymakers and pursues two general goals. First, we aim to illuminate the fairness dilemma of aircraft noise exposure using the psychological perspective of social justice research. In order to better understand the psychological underpinnings of how individuals deal with the fairness dilemma regarding noise exposure, empirical research on the psychology of social justice offers some valuable insights. Social justice research distinguishes between different forms of fairness, namely, distributional fairness, procedural fairness, and informational or interactional fairness. All three conceptualizations provide unique and illustrative approaches that can inform the understanding of how unfairness is perceived in the context of noise distribution. However, insights from social justice research cannot only be used to better describe and explain the behavior of angry and annoyed residents, which is often expressed in complaints and protest [43]. They also provide empirically based starting points for developing communicative interventions in order to enhance the residents’ perceived fairness of the noise distribution and, thus, to manage the acceptance of the airport as a neighbor, noise annoyance, and the burden of some residents from an intervention perspective. Our second goal is to emphasize and review possible ways to resolve, or at least handle, the emotional and attitudinal consequences of noise distributions that are perceived as unfair. The paper concludes by developing recommendations of how airports can implement these fairness psychological findings in practical terms.

## 2. Distributive Fairness

In this section, we outline and discuss theoretical explanations for why and how the distribution of aircraft noise is perceived as a fairness issue. We present empirical research and derive intervention strategies.

### 2.1. What Is a Fair Noise Distribution?

As argued before, aircraft noise annoyance can result from a social conflict over distributional fairness. However, we also consider when and how individuals perceive distributions to be unfair. Equity theory [44] and relative deprivation theory [45] provide similar answers to this question.

Both approaches emphasize that humans evaluate fairness based on social comparisons of cost–benefit ratios. According to equity theory [44], distributive fairness or equity is experienced when the cost–benefit ratios between individuals are perceived as equivalent. Contrarily, when inequality between these ratios is perceived, individuals tend to feel that they are being treated unfairly. In a similar vein, relative deprivation theory posits that anger and rumination are triggered when individuals perceive themselves to be deprived relative to what they expect to deserve or relative to what significant others receive (for an overview, see [46]).

Leventhal [47] extended these lines of research by showing that individuals evaluate deservingness and fairness based on different principles. Besides the equity rule as described above, he also suggests the equality and the need rule. The equality rule suggests that any costs or benefits should be distributed equally among all those people that are eligible. The needs rule takes into consideration the vulnerability of individuals. It suggests that vulnerable people (such as children, sick or old people) should be less exposed to costs or burdens and benefit more easily than healthy adults. A further type of distribution principle is based on Bentham’s utilitarianism [48], according to which costs or benefits should be distributed in such a way that the greatest possible overall benefit would be generated.

Summarizing these different principles, noise could be distributed in several ways:

1. The aircraft noise is distributed in a way that the ratio between the disadvantages (i.e., the burden of the noise exposure) and the benefits of the nearby airport are equal between all residents (equity rule/contribution rule);
2. Noise should be distributed equally over as many residents as possible, regardless of the composition of residents and other environmental strains (equality rule);
3. Residents with special needs (e.g., children, sick or elderly) should be protected from the noise as much as possible (needs rule);
4. Noise should be distributed in such a way that the highest number of residents will be protected from noise, even if some residents will experience very high levels of noise (utilitarianism approach).

Unfortunately, no answer can be given at present to the question of which of the presented principles of distribution of aircraft noise should now be implemented in order to achieve the fairest perception of aircraft noise. In a preliminary study [49], no significant effect of the different allocation principles on the perceived fairness was found. However, future research should address this issue. An example of how noise distribution is technically modifiable can be shown in a statistical evaluation of flight operational characteristics that points out that by reorganizing the departure direction and/or departure performance restrictions, noise could be distributed in order to minimize the noise impact in certain areas [50]. New technologies, such as the automatic dependent surveillance-broadcast (ADS-B) may be beneficial by providing more accurate data than conventional radar systems on, e.g., take-off ground run distance and altitude data [51].

Individuals differ in their fairness evaluations not only because they rely on different fairness principles but also because they differ in their general sensitivity to perceiving and experiencing unfairness [52]. Based on these findings, some people experience stronger emotional reactions to injustice (i.e., anger and outrage) and are more likely to ruminate on perceived unfairness. These individual differences can partly explain why some people are more engaged in political protest and opposition towards large public transport projects [13].

This research on distributional fairness consequently suggests two different strategies for interventions to minimize perceived unfairness in the distribution of airport noise. The first strategy is to implement the fairness principle that is most likely to be perceived as fair by a respective group of residents. The second strategy is to compensate individuals who are disadvantaged by a specific noise distribution so that their ratio between costs and benefits improves.

### 2.2. Finding the Balance—Compensation to Amend for an Unfair Distribution?

A cost–benefit balance can either be achieved by reducing the cost (i.e., the individual noise level) or by increasing the benefit of individuals who are affected by aircraft noise. The following interventions focus mainly on the reduction of individual costs. However, potential interventions increasing the individual benefits will also be discussed as these have not yet been implemented.

#### 2.2.1. Noise Insulation

Noise insulation and other abatement measures at home (i.e., insulation of the wall, soundproof windows with or without a ventilation system) can drastically reduce indoor sound levels, potentially reducing noise. A telephone survey in 2010 with citizens in proximity to Frankfurt Airport revealed that a large proportion of the residents do not claim insulation entitlement, and, when they do so, they often do not use the ventilation system during the night. Schreckenberg [53] highlights that noise insulation measures at home lack efficiency in order to reduce aversive noise effects such as annoyance.

These findings emphasize that the interventions that seem to be the most important often do not lead to the desired result in reducing annoyance. Schreckenberg [53] points out that while insulation has the potential to drastically reduce indoor noise levels, it cannot replace active noise control measures.

#### 2.2.2. Providing Noise-Free Times

One example of a noise control measure which is currently studied in the vicinity of Heathrow Airport is the so-called noise respite [54]. The development of satellite navigation technology allows an aircraft to fly more accurately on specified paths (performance-based navigation, PBN), resulting in a greater control over the noise distribution. The idea of noise respite is that flight paths are varied so that residents can enjoy noise-free times, while other residents experience more noise at the same time, and vice versa. By doing so, the aircraft noise can be shared across communities so that some communities experience respite. While there is currently neither a clear and consistent definition of respite nor of the duration of noise-free times to be perceived as respite, we note that relief can be defined as a break from or a reduction in aircraft noise. In contrast, respite can be defined as scheduled relief from aircraft noise for a period of time. The Respite Working Group currently claims that small changes in noise (i.e., 2–3 dB) are hardly noticed by residents. Residents would perceive an increase in noise rather than a decrease of similar magnitude [54].

At Frankfurt Airport, the concept of respite was also imposed in 2015, about 3.5 years after the opening of the fourth runway (October 2011) with temporary closure of two of the four runways in the shoulder hours [55], which are the hours immediately before and after the night. In between these shoulder hours, from 11 p.m. to 5 a.m., there has already been a night curfew implemented a Frankfurt Airport since November 2011. Within these shoulder hours, this results in a decrease in exposure for some residential areas and an increase in other areas. In summary, however, this led to more residents being relieved from aircraft noise rather than being additionally burdened by it for the whole night from 10 p.m. to 6 a.m. Focus groups and a telephone survey revealed that these respite operations exert only marginal effects on the perception, and residents also hardly noticed any difference, perhaps because of the fact that aircraft sounds were still audible (though softer) in the shoulder hours. Moreover, only a minority of participants were informed about the operations being implemented. Nevertheless, participants were in favor of a continuation of the respite operations at Frankfurt Airport [55].

#### 2.2.3. Compensating Loss of Value

Another approach that refers to distributive fairness aspects is the financial compensation for the loss of value of real estate which had been observed, e.g., [56]. Buying up the properties by the airport is a measure that is already being practiced as, for instance, in certain districts at Frankfurt Airport [57] after the opening of the new runway, and as is currently the case at Zurich Airport [58]. Additionally, Heathrow Airport announced compulsory purchases in the case of an additional runway being built [59]. Further compensation programs grant owners a one-time monetary compensation for the impaired opportunity to use the outdoor living areas of a dwelling, as is already part of, for instance, the act for protection against aircraft noise [60] and that of Dusseldorf Airport or Airport Berlin Brandenburg. Despite those efforts, systematic and empirical evidence of its effectiveness in, e.g., reducing annoyance has not been reported yet.

#### 2.2.4. Increasing Individual Benefits

**Figure 1:**
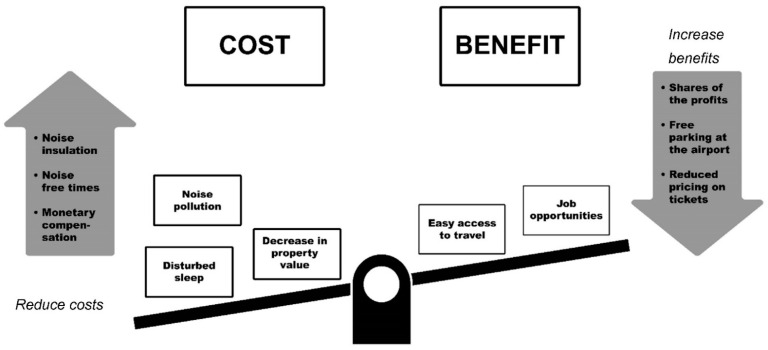
Illustration of balancing the individual cost–benefit ratio.

Whilst interventions focusing on the “reducing cost” part of the distributive fairness aspects have already been implemented, such as the ones mentioned above, interventions relating to increasing the individual benefits (of the airport) were, to the best of our knowledge, not yet introduced. To act in line with the social exchange theory [44], one would suggest that interventions focusing on increasing the individual benefit also contribute to the perception of fair distribution. As depicted in Figure 1, the overall goal is to balance the cost–benefit ratio of residents. Besides the already mentioned interventions to reduce the individual costs, examples of interventions to increase the personal benefit, which were derived from a focus group study performed around Cologne-Bonn airport [61], would be:

- Providing shares of the profits from the airport;
- Free parking at airports;
- Reduced pricing on flight tickets.

These exemplary interventions to increase individual benefits while being exposed to aircraft noise have been neither implemented nor evaluated, so no assertion can be made about the effectiveness.

Social justice research has revealed that people not only focus on the outcome when they judge the fairness of a distribution; whether people perceive themselves being treated fairly or unfairly is also determined by features of the process, i.e., how the distribution was decided and communicated.

## 3. Procedural Fairness

Since the 1970s, a substantial amount of research indicates that the perceived fairness of a distribution is strongly affected by the procedures that are used to decide the distribution [62,63,64]. The so-called fair process effect is one of the most prominent findings in this context [62,65]. It indicates that when participants have a voice in an allocation decision, they perceive the outcome as fairer and evaluate it more positively, irrespective of the final distribution. Research on the fair process effect suggests that a fair process might have more influence on the overall fairness evaluation than the outcome itself [62]. This section summarizes the findings from research on procedural fairness and describes how these findings can be used in the area of aircraft noise allocation.

### 3.1. What Is Procedural Fairness?

As described, it was becoming increasingly clear that people are not only concerned with the outcomes of a decision, but, more importantly, with the procedures that lead to the decision. Thibaut and Walker [63] first examined procedural fairness in situations when a decision was made by a third party or legal authority that had consequences for an individual. The authors argued that a procedure is perceived as fairer when the individual has some amount of control in the decision-making process. They distinguished between process control and decision control. Process control means that an individual has the chance to express his or her perspectives and to bring arguments before the decision is made. Decision control, in contrast, refers to the actual amount of influence the individual has on the decision-making process.

The importance of procedural aspects can be explained via the theoretical framework of Stallen [34].

Procedural fairness can be seen in this context as a coping possibility because it fulfils different psychological needs. Procedural fairness is important for people because it conveys information about one’s status in the group. Being granted some amount of control in the process of a decision implies that one is a valued member of a group and thus enhances the feeling of belonging and self-esteem [62,64]. Information about procedural fairness is also known to heuristically reduce uncertainty in social relationships [66]. Thus, perceived procedural fairness is used as an indicator of trustworthiness when people want to reduce the uncertainty of not knowing if an individual or a party can be trusted (see [67,68,69]). Essentially, a fair procedure operates psychologically in a similar fashion to stereotypes; thus, it helps humans to reduce uncertainty in a fast and frugal manner and is therefore assumed to reduce stress [65,66]. In line with these notions, research in the occupational and legal context revealed that fair procedures have an impact on pay evaluations [65,70]; job satisfaction; trust in the management, e.g., [71] organizational commitment, and job performance [72]; and on attitude towards judges and the court [73]. Nonetheless, how can procedural fairness be achieved?

### 3.2. Characteristics of Procedural Fairness

Leventhal [47] developed six criteria for procedural fairness (listed below) which were mainly examined in the context of organizational psychology. A meta-analytic review indicates that the combination of these criteria is a better predictor of perceived procedural fairness than process control alone [72]. The application of these theoretical considerations from Leventhal [47] in the context of the aircraft noise problems leads to the following recommendations on how procedures that determine noise-related decisions should be designed:

1. Representativeness: During all phases of decision-making procedures (e.g., the opening of a new runway), the concerns and opinions of all affected citizen should be represented. This could, for example, be carried out via an open hearing or by having representatives for each party. This picks up the idea of giving residents a “voice”.
2. Consistency rule: Procedures are consistent across residents. In other words, the criteria for when and how an airport pays for noise insulation or compensation measures are transparent and applied coherently for every resident; nobody is given an advantage or disadvantage.
3. Bias suppression rule: Decisions by the airport or airport stakeholders should not be taken solely for self-interest and economic reasons, although the operation of an airport is initially exclusively economic in nature. For example, noise thresholds and thus decisions to ban night flights or certain loud aircrafts should be based on scientific knowledge of health effects. To prevent decisions based on self-interest, neutral bodies such as ombudsmen should be involved.
4. Accuracy rule: The allocative process is based on sufficient, correct, and appropriate information. In this case, e.g., noise insulation schemes should be based on the most recent scientific data about the impact of noise on health.
5. Correctability rule: Opportunities exist to alter or reverse an inaccurate decision at various stages of a process. Accordingly, all parties involved in this process have the chance to appeal or challenge a decision. This should imply that, e.g., night flight permissions should be revoked if new insights on the effect of nocturnal noise and noise-induced sleep disturbance on health outcomes are obtained. If decisions are made that affect the citizens concerned, they should be reconsidered and adapted accordingly in light of newer knowledge.
6. Ethicality rule: Processes that lead to a certain noise distribution should generally be in line with fundamental ethical and moral standards. In concrete terms, this means that decisions on noise distribution should be approved by, for example, an ethics committee. An ethics committee could surveil whether sub-populations are treated equally or whether the noise distribution is associated, for instance, with the socio-economic status of the residents of noise-exposed areas. Moreover, it can decide, for example, to appeal against the night flights at an airport if the recent research on the effects of noise at night reveals that lasting damage can be caused to the affected inhabitants.

In summary, procedural fairness research provides an understanding of the boundary conditions in which decisions about distribution and exposure to aircraft noise are more readily accepted by the public and met with less resistance. The empirical evidence on the role of procedural fairness in the evaluation of noise distribution should also be considered.

### 3.3. The Benefit of Fair Procedures in the Distribution of Aircraft Noise Exposure

The contribution of perceived process and decision control to annoyance judgments was studied experimentally in the laboratory. Maris, Stallen, Steensma and Vermunt [74] ran two laboratory experiments that focused on voice and process control as one aspect of procedural fairness. In the first experiment, the participants who could voice their preference for a certain sound (i.e., bird song, radio sound or aircraft sound) and who believed that this preference was considered (fair procedure) were less annoyed by aircraft noise than participants who could not voice their preference (neutral procedure) and who were also exposed to an aircraft sound. In the second experiment, it was shown that participants who voiced their preference and whose preference was ignored (unfair procedure) were significantly more annoyed than participants who could not voice a preference (neutral procedure).

Moreover, a survey with airport residents [75] showed that residents who think that “the opinion of all the citizens directly affected by the airport will make a difference in the decisions about the airport and the area surrounding it” (p. 341) are less annoyed than residents who disagreed with this statement.

In fictive airport expansion scenarios that differed, among other things, in the amount of participation opportunities for residents (few or manifold), it was shown that when offered manifold participation opportunities, subjects around two European airports exhibited more social acceptance of the (fictional) expansion plans than when only given few participation opportunities [49].

In a field study within the NORAH project, which was a multidisciplinary research project examining the effects of noise on annoyance, cognition and health, Schreckenberg and colleagues [76] performed a sensitivity analysis and concluded that considerable differences between annoyance ratings depended on the procedural fairness perception of residents.

Thus, when decisions are being taken regarding airport expansion plans, new take-off and landing procedures, a prolongation of operations at night or the establishment of a runway alternation system, it is important to create a framework that makes use of fairness–psychological findings, enhancing the probability of these being perceived as a just course of action. As it could be shown, a fair procedure offers the possibility of dealing with the necessarily unfair distribution of aircraft noise.

However, it has to be stated clearly that this does not mean that the distribution of noise over the population has no influence on the perception of fairness, but the framework conditions leading to this certain distribution could be created in such a way that it essentially influences subjective fairness assessments. Cohen [77] already voiced concerns in 1985 about the potential abuse of procedural fairness as it enhances the subjective feeling of fairness, even though the objective criteria could be patently unfair.

### 3.4. From Theory to Practice—Incorporating Procedural Fairness Aspects in Aircraft Noise Management

Aircraft noise interventions have mostly focused on the mitigation of noise at the source as well as operational restrictions and land-use planning in accordance with the balanced approach to aircraft noise management suggested by the International Civil Aviation Organization (ICAO). However, the importance of including residents’ perspectives in a balanced approach to aircraft noise management was recently underlined by the ICAO (2017). Measures such as providing an opportunity for residents to give feedback and express their views which meet the crucial criteria of procedural fairness have been recommended. However, the degree to which airports carry out such participatory communication is limited, and evaluations of the benefit of such measures almost never happen [78].

In contrast, mistrust between the opposing parties in airport-related decision making, the impossibility for the voice of affected residents to be heard, and the lack of transparency of the airport operators, thereby fostering protests and mobilization against an airport expansion, was demonstrated [79]. In this case study from Barcelona Airport, residents were given false promises about the noise generated by the construction of a new runway and the use of certain configurations, which made it impossible to predict the time and level of noise exposure. In this example, citizens affected by the noise proposed technical solutions that reduced the noise in those areas while simultaneously allowing the airport to operate properly. The proposal made by the citizens was very effective in reducing noise levels by 15–20 dB L_eq_ and brought a period of peace between the airport and affected residents.

Reviewing different case studies in regard to communication and engagement efforts conducted by European airports, Heyes [78] identified Heathrow, Vienna and Frankfurt as those where the airport does not take the dominant role as the “expert”. The Vienna Dialogue Forum offers the only real opportunity in the vicinity of an EU airport for a real two-way exchange, providing all participants with a voice. The Vienna Dialogue Forum is the most extensive mediation process in Europe, which was implemented with the planning of the third runway at Vienna Airport. In this mediation process, around 50 interested parties, such as action groups and neighborhood communities, are working together to find solutions that would be acceptable for all involved parties [80].

All parties are represented in the process; this the Vienna Dialogue Forum cares for consistent procedures, bias is suppressed since the airport is not the one in charge, and the results should be based on accurate and ethical considerations. Although this seems to be a good example of how procedural fairness can be implemented in the context of aircraft noise politics, its impact has not yet been evaluated.

In the context of respite, Porter [54] also pointed out that for respite to be really helpful for communities, it is important to engage all communities during all phases of respite design and implementation. Recently, Porter [54] explained that more effective and successful implementations of respite have proactively engaged and consulted with the local communities.

## 4. Informational and Interpersonal Fairness

Research from justice and organizational psychology has shown that fair procedures including opportunities to have one’s voice heard do not suffice for the feeling of being fairly treated. Instead, the quality of interaction between the involved parties, i.e., the way these decisions are communicated to the affected people, also matters [81]. In this final part of the paper, we present fairness aspects regarding information provision and interaction between the airport and residents and discuss why these aspects are important.

### 4.1. What Is Informational and Interpersonal Fairness?

Bies and Moag [82] introduced the construct of interactional justice which focuses on the quality of the interaction between decision-makers and individuals that are affected by these decisions [69]. Subsequently, this concept has been further differentiated into interpersonal fairness and informational fairness. Interpersonal fairness focuses on the degree to which people are treated with politeness, dignity, and respect by the decision-making party [43]. Informational fairness describes the quality of the explanations given to the affected people that justify the reason for the application of a certain decision-making procedure or the distribution of the outcome in a certain way [43].

In other words, communication management of the airport should not only provide engagement opportunities for residents but also communicate in a way that is perceived as fair. Sommerfeld [83] provided empirical evidence for this assumption. Based on qualitative interviews, she concluded that residents ask for better, i.e., more comprehensive and transparent, communication and information in terms of creating a better relationship with the airport. In a similar vein, other studies revealed that residents most often desired honest and comprehensive information when asked what the airport could do to achieve and maintain good neighborliness, [84,85]. Moreover, Maziul and Vogt [85] argued that the introduction of a free-toll telephone service that enables residents to receive aviation-related information might be able to reduce community annoyance.

### 4.2. How to Create a Fair Interaction between the Airport and Its Residents?

Bias and Moag [82] focused on fairness in terms of interpersonal treatment on the one hand and communication and information on the other hand and postulated four criteria that people use to evaluate interpersonal fairness, which can be applied easily to the aircraft noise context:

Criteria of informational fairness:

1. Truthfulness: Communication from the airport should be made in an honest and candid way. This means that residents must be informed about the scope, duration and level of noise during the decision-making processes [86]. This form of truthfulness stands in conflict with a strategy of downplaying potential burdens of noise exposure to avoid protests and complaints by the affected residents. From the residents’ perspective, this strategy might be understood as a kind of deception, especially when the claims ultimately prove false.
2. Justification: Decisions regarding noise exposure are perceived as fairer when an adequate justification or reasoning is provided [87], for example, when objectives and intentions are honestly and openly explained. The timing of justification also matters. When decisions are made about aircraft noise, the final outcome is seen as fairer if information about the process is given in advance than if it is given after the outcome has been determined. This implies that information should always be provided as early as possible [67].

Criteria of interpersonal fairness:

3. Respect: The interaction should be respectful and polite, i.e., the airport should treat the affected citizens with respect. All subjective feelings must be taken seriously, and residents should be encouraged to actively participate in the decision-making process. The airport should emphasize the relevance of each resident and listen to their feelings and perceptions.
4. Propriety: Prejudicial and improper comments are avoided, even when dealing with enraged citizens. Even when interacting with very angry residents, responsible contact persons must be friendly, polite, and courteous at all times. It is important to understand that residents may be emotional and heated and, therefore, sometimes behave in an unfriendly manner.

### 4.3. Setting the Right Tone—Interactional Justice in Practice

In the context of respite, the working group of Porter [54] found out that good communication and transparent engagement is key for the successful implementation of respite programs.

Additionally, an example of how informational fairness can be beneficial is the study conducted by Schreckenberg [88]. In two geographically separated study areas along a railway line in south Germany, the impact of railway grinding on residents’ responses to railway noise was investigated. Rail grinding was applied on this railway line, a measure to reduce the roughness of rail surfaces with the consequence of lower noise levels emitted by contact of the wheel on the railroads. Despite having almost zero effect on the overall noise level reduction, in the study area in which residents were informed about the grinding and its noise-reducing effect prior to the intervention, participants reported lower levels of annoyance and disturbances afterwards than before, whereas in the uninformed area, no change in noise disturbance and annoyance responses was observed. The message of this study is not to issue press releases of fictitious interventions, but rather to highlight that providing information about interventions can enhance its effectiveness.

Furthermore, Hooper and Flindell [89] draw conclusions on the results of recent and mainly qualitative research and claim that residents often believe that authorities have no real interest in communicating at eye level with the public as current information is “too complicated, over-technical and does not even focus on information which the public actually want or need to know” [89]. The authors point out that providing understandable information to residents could make them more tolerant and accepting because it increases understanding and allows residents to identify and focus on real issues of importance, but also the limitations of what can be done. Gasco and colleagues have also found this to be the case, as currently, the communication of information on noise is too technical to be understood by lay people, certain standards are lacking to compare noise between airports, the measurement points are sometimes incomplete and do not reflect the actual noise nuisance and, finally, little feedback from affected citizens is seen and incorporated into the information preparation [90].

A good example of interactional fairness is represented by the Vienna Dialogue Forum since this mediation process is based on mutual respect and propriety [91]. Approximately 50 affected parties (both neighborhood associations and interest groups) are represented at eye level in this mediation process. Necessary information is provided in a transparent and truthful manner in order to arrive at decisions that are appropriate and acceptable to all concerned.

As Flindell pointed out, “(qualitative research could reveal that) many residents will tolerate being annoyed from time to time if they also understand what has been done to reduce the problem and why the remaining annoyance is unavoidable. (But the) airports (need) to fully engage with their surrounding communities to explain and justify (in a respectful and polite manner) where noise is unavoidable and to make their economic and social contributions to general welfare much more explicit” [92].

### 4.4. Recommendations for Practical Implementation

In this review, we outlined the dilemma of fairness in the distribution of aircraft noise from the perspective of fairness research. As the accessibility of airports is generally not only necessary but even desirable, noise has to be distributed over a certain airport region, which will ultimately put more strain on some residents compared to others. By taking different perspectives on the psychology of fairness into consideration, we argue that such an overall unfair event, such as the distribution of noise, can still be perceived as more or less unfair. In Table 1, we summarize the findings of this review and suggest concrete applications of fairness principles in the context of planning strategies and communication management of airports and describe to what extent they seem feasible to implement. It is important to note that these are the opinions of the authors, as empirical evidence is scarce.

## 5. Conclusions and Outlook

This paper had two main objectives. First, we aimed to analyze the fairness dilemma of aircraft noise distribution from the perspective of social justice research. Second, we aimed to identify ways of dealing with this unfair distribution of noise and provide recommendations on how to implement fairness considerations in concrete interventions to reduce annoyance from aircraft noise.

The fairness dilemma results from the practical problem that aircraft noise is generally shouldered by a group of residents, while the benefits of the airport are shared by all residents. We outlined and discussed different forms of fairness, namely, distributional fairness, procedural fairness and informational or interactional fairness, as distinguished in the scientific literature on the psychology of justice. All of these different perspectives provide unique and valuable insights on how to reduce perceived unfairness in the process of noise distribution. However, the empirical evidence on fairness perception by residents in the field of aircraft noise reduction is scarce. Precisely because the empirical evidence base is so small, future research is of great importance. Unfortunately, there are currently no established psychometric questionnaires available to measure fairness perception in the context of noise research in a valid and reliable way. Thus, there is a need for the development of psychometric instruments. In order to gain a deeper understanding of the nature of fairness evaluations, qualitative studies (e.g., focus groups) should also be conducted with airport residents.

We outlined existing interventions in the field of aircraft noise distribution that have considered specific aspects of fairness research. However, the theoretical principles have not been applied systematically in the past. Therefore, we derived concrete recommendations from social justice research on how perceived fairness in the distribution of aircraft noise can be increased. These recommendations are summarized in Table 1. It must be noted that these practical recommendations are exemplary and might not cover the full range of potential applications. Importantly, there is a lack of empirical evidence on how capable these interventions are at reducing the impact of the fairness dilemma on the perceived annoyance from noise. Future research is needed to systematically evaluate the effectiveness of such fairness interventions. Many of these recommendations require additional effort on the part of agents in airport management and communication. However, research in other areas (e.g., organizational justice) provides us with confidence that these efforts can improve the effectiveness of other measures to reduce annoyance from aircraft noise and increase the acceptance of the airport and local aviation.

## Figures and Tables

**Table 1:** Summary and practical implementation of fairness research.

Fairness Category	Fairness Aspect	Application	Feasibility
Distributive fairness	Creating fair noise distribution	Distribute noise (a) to protect residents with special needs (children, sick or elderly), (b) equally over as many residents as possible, (c) in such a way that the highest number of residents will be protected.	Little empirical evidence on which principle of distributive justice is perceived as fairest by residents.
	Improving the individual cost–benefit ratio by:		
	(a) Reducing noise and noise-related burden	Noise insulation, providing noise-free times and spaces, compensation (buying up properties, providing monetary compensation).	Noise reduction interventions are often regulated by national law. However, there is a lack of empirical evidence on which of the implemented interventions is (most) effective.
	(b) Increasing individual benefit	Providing shares of the profits from the airport, free parking at airports or reduced pricing on tickets.	New type of intervention derived from the literature presented here. However, there are no empirical studies on this.
Procedural fairness	Bias suppression	Decisions taken by the airport are not exclusively led by self-interest and economic reasons.	Involving independent and neutral bodies (e.g., ombudsmen) could be an important component of a fair decision-making procedure.
	Representativeness	Provide opportunities and ensure opinions of affected residents are represented during all phases of the decision making.	The Vienna Dialogue Forum can be a model for the implementation.
	Consistency	Procedures are kept consistent between residents (e.g., criteria for noise insulation, noise protection zones).	Legislation is not the same throughout the country, so consistent treatment of all citizens is difficult to implement.
	Accuracy	Decisions regarding noise distribution should be based on sufficient, correct, and appropriate information.	By including scientific advisors, this step should be easily applicable.
	Correctability	Affected parties have the opportunity to challenge a decision (e.g., night flight permissions)	Affected persons should be able to challenge any decisions at any time. However, this would require a change in legislation and is therefore rather difficult to achieve.
Informational fairness	Truthfulness	Communicating to residents in an honest, transparent, and candid way. Informing about the impact of change (e.g., opening a new runway) has to be truthful, exhaustive, and understandable, even when communicating negative news.	This point is both simple to implement and effective.
	Justification	Justifying, e.g., the decision to build a new runway comprehensively, in a timely manner and in a language that laymen understand so that relevant information is not “hidden” behind technical jargon and abstract noise exposure metrics.	Creating a resident-oriented communication can be achieved without any further costs but requires understandable metrics.
Interpersonal fairness	Propriety	Avoid uncivil behavior and prejudicial and improper comments. Every interaction, even with angry residents, has to be impartial and polite.	Communicators can be trained to deal with residents, and this should be easily implemented.
	Respect	Every interaction should be respectful and polite. Respect residents’ feelings and perceptions and encourage active engagement in the decision-making process.	Same as for propriety
